# Analysis of BAC-end sequences (BESs) and development of BES-SSR markers for genetic mapping and hybrid purity assessment in pigeonpea (*Cajanus *spp.)

**DOI:** 10.1186/1471-2229-11-56

**Published:** 2011-03-29

**Authors:** Abhishek Bohra, Anuja Dubey, Rachit K Saxena, R Varma Penmetsa, KN Poornima, Naresh Kumar, Andrew D Farmer, Gudipati Srivani, Hari D Upadhyaya, Ragini Gothalwal, S Ramesh, Dhiraj Singh, Kulbhushan Saxena, PB Kavi Kishor, Nagendra K Singh, Christopher D Town, Gregory D May, Douglas R Cook, Rajeev K Varshney

**Affiliations:** 1 International Crops Research Institute for the Semi-Arid Tropics (ICRISAT), Patancheru, Hyderabad, 502324, India; 2 Department of Genetics, Osmania University, Hyderabad 500007, India; 3 Department of Biotechnology and Bioinformatics Centre, Barkatullah University, Bhopal 462026, India; 4 Department of Plant Pathology, University of California, Davis, CA 95616, USA; 5 Department of Biotechnology, University of Agricultural Sciences (UAS), Bangalore 560065, India; 6 Department of Plant Breeding and Genetics, CCS Haryana Agricultural University (CCSHAU), Hisar 125004, India; 7 National Center for Genome Resources (NCGR), Santa Fe, N M 87505, USA; 8 National Research Center on Plant Biotechnology (NRCPB), New Delhi 110012, India; 9 J. Craig Venter Institute (JCVI), Rockville, MD 20850, USA; 10 Generation Challenge Programme (GCP), c/o CIMMYT, 06600 Mexico DF, Mexico

## Abstract

**Background:**

Pigeonpea [*Cajanus cajan *(L.) Millsp.] is an important legume crop of rainfed agriculture. Despite of concerted research efforts directed to pigeonpea improvement, stagnated productivity of pigeonpea during last several decades may be accounted to prevalence of various biotic and abiotic constraints and the situation is exacerbated by availability of inadequate genomic resources to undertake any molecular breeding programme for accelerated crop improvement. With the objective of enhancing genomic resources for pigeonpea, this study reports for the first time, large scale development of SSR markers from BAC-end sequences and their subsequent use for genetic mapping and hybridity testing in pigeonpea.

**Results:**

A set of 88,860 BAC (bacterial artificial chromosome)-end sequences (BESs) were generated after constructing two BAC libraries by using *Hin*dIII (34,560 clones) and *Bam*HI (34,560 clones) restriction enzymes. Clustering based on sequence identity of BESs yielded a set of >52K non-redundant sequences, comprising 35 Mbp or >4% of the pigeonpea genome. These sequences were analyzed to develop annotation lists and subdivide the BESs into genome fractions (e.g., genes, retroelements, transpons and non-annotated sequences). Parallel analysis of BESs for microsatellites or simple sequence repeats (SSRs) identified 18,149 SSRs, from which a set of 6,212 SSRs were selected for further analysis. A total of 3,072 novel SSR primer pairs were synthesized and tested for length polymorphism on a set of 22 parental genotypes of 13 mapping populations segregating for traits of interest. In total, we identified 842 polymorphic SSR markers that will have utility in pigeonpea improvement. Based on these markers, the *first *SSR-based genetic map comprising of 239 loci was developed for this previously uncharacterized genome. Utility of developed SSR markers was also demonstrated by identifying a set of 42 markers each for two hybrids (ICPH 2671 and ICPH 2438) for genetic purity assessment in commercial hybrid breeding programme.

**Conclusion:**

In summary, while BAC libraries and BESs should be useful for genomics studies, BES-SSR markers, and the genetic map should be very useful for linking the genetic map with a future physical map as well as for molecular breeding in pigeonpea.

## Background

Pigeonpea [*Cajanus cajan *(L.) Millsp.], also known as tuar or arhar, is an economically important legume crop with an annual production of 3.65 Mt. Cultivation of pigeonpea occurs on ~5 million hectares, primarily in Asia and countries of eastern and southern Africa, and to a lesser extent in countries of Latin America and the Caribbean. As a member of the sub tribe *Cajaninae*, pigeonpea is contained in an early diverging lineage of tribe *Phaseoleae*, a monophyletic group of legumes that contains several of the world's most important food legumes including soybean, common bean, cowpea and mung bean. Similar to most other *Phaseoleae *species, pigeonpea contains 11 pairs of chromosomes (2n = 22) and has a moderately sized genome in the range of 0.853 pg or 858 Mbp [1].

India is the world's largest producer of pigeonpea and the presumed center of origin [2]. Relative to most other crop legumes pigeonpea is highly drought tolerant, being able to retain productivity with less than 650 mm annual rainfall. Owing to its capacity for symbiotic nitrogen fixation, pigeonpea seeds have high levels of protein and they specifically enriched for amino acids that are often limiting in the human diet, including methionine, lysine, and tryptophan. In resource poor areas of the world, pigeonpea serves as an important forage and cover crop, while the stems provide wood for tool making and fuel, and thatch for roofing. These factors, especially the ability to withstand elevated temperatures and limited water availability, add to pigeonpea's importance as a crop in semi-arid tropical (SAT) regions of the world, especially in the SAT of India where approximately 77% of global production occurs. Despite its importance in the SAT regions, little concerted research effort has been directed at either improvement or technology transfer in this crop. Thus, the pigeonpea production has remained static [3] and a range of biotic and abiotic stresses continue reduce yields by 50% or greater [4]. Among the most important limiting factors are *Fusarium *wilt, sterility mosaic disease, pod borer, soil salinity and water logging. Very recently, hybrid breeding technology based on the cytoplasmic-nuclear male-sterility (CMS) system has been implemented in the pigeonpea breeding programme at ICRISAT [5], and this technology holds great potential to increase pigeonpea productivity.

Various advances in plant biotechnology and especially genomics together with traditional plant breeding technologies have led to the development of new improved varieties in a number of crop species with greater tolerance/resistance and higher yield [6,7]. In this context, molecular markers play a very important role as these are used for estimating diversity in germplasm, trait mapping, molecular breeding, genetic purity assessment of hybrid seeds, etc. Among a range of molecular markers starting with isozymes, RFLP (restriction fragment length polymorphism), RAPD (random amplified polymorphic DNA), AFLP (amplified fragment length polymorphism), SSR (simple sequence repeat), DArT (diversity array technology), and most recently SNP (single nucleotide polymorphism), that have become available during last two decades [8], SSR markers have emerged as the current markers of choice for plant genetics and breeding applications [9]. While SNP markers have a promising future in plant breeding applications, and may augment or displace SSR based marker systems, SNP based markers and associated technologies are in their infancy in most crops, including pigeonpea, while SSR marker technologies are better established for wide spread use in molecular breeding.

In case of pigeonpea, at present, only a few hundred SSR markers are available [10-13], a situation that is further hampered by low levels of genetic diversity within cultivated germplasm demands development of SSR markers at large scale.

Traditionally, three approaches are used for identification and development of SSR markers: (i) construction of SSR-enriched library followed by sequencing of SSR positive clones [9], (ii) mining of EST (expressed sequence tag) transcript sequence generated by Sanger sequencing [14] or short transcript sequences generated by next generation sequencing technologies [15], (iii) mining the BAC (bacterial artificial chromosome)- end sequences (BESs) [16]. So far, the first two approaches have been used for developing SSR markers in pigeonpea with some success despite the labour-intensive and time consuming nature of the SSR enrichment and very low polymorphism levels of SSRs identified from the mining of transcript sequences. The development of SSR markers from BESs circumvents the limitations of the first two approaches, as a large number of SSRs can be rapidly identified and such genomic SSRs tend to display higher level of polymorphism relative to transcript associated SSRs. In addition, BES-SSR markers serve a useful resource for integrating genetic and physical maps [16-18].

The present study was undertaken with following objectives: (i) construction of two BAC libraries and sequencing of BAC-ends, (ii) comprehensive analysis of BAC-end sequences (BESs) for gaining insights in pigeonpea genome, (iii) mining the BESs for development of large scale SSR markers, (iv) characterization of newly developed BES-SSR markers on a panel of parental genotypes, (v) development of the *first *SSR-based genetic map for pigeonpea, and (vi) identification of an informative set of SSR markers suitable for purity assessment of two leading hybrids, ICPH 2438 and ICPH 2671 to facilitate efficient hybrid seed production.

## Results

### BAC-end sequence analysis

Two BAC libraries were developed from pigeonpea cultivar "Asha", based on partial digestion with *Hin*dIII and *Bam*HI restriction enzymes. BAC clones were sequenced from both insert ends to yield 88,860 DNA sequences with an average read length of 620 bp.

**Figure 1:**
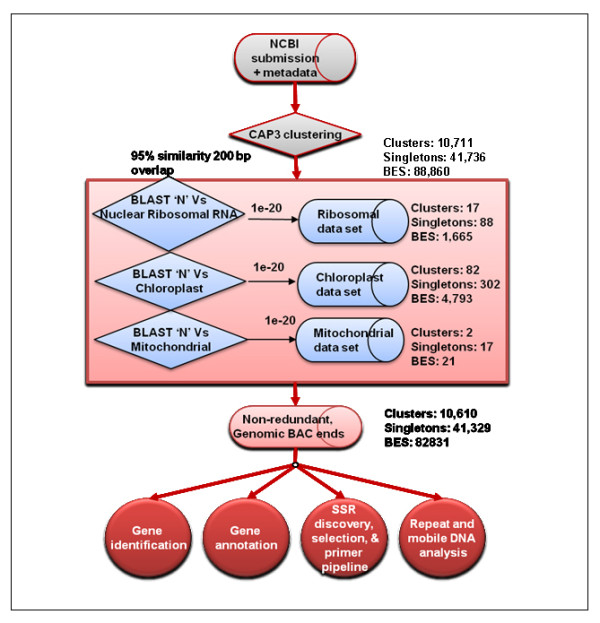
**Annotation pipeline for analysis of BESs**. This pipeline resulted in selection of non-redundant genomic BAC-ends which excluded organeller sequences, and further identification, annotation of non-redundant sequences together with SSR discovery, selection and primer designing.

As a prelude to the comprehensive analysis of BAC-end sequences, we analyzed BESs for redundancy between clones and for sequence content as well as for removal of cytoplasmic organellar sequences using the annotation pipeline shown in Figure 1. Sequences were clustered using criteria of ≥95% identity and ≥200 bp overlap, producing a set of 41,736 singleton sequences and 10,711 sequence clusters. This non-redundant sequence set was filtered for rRNA, chloroplast and mitochondrial sequences using BLAST'N' against datasets of the corresponding sequence types, yielding a set of 41,329 singletons and 10,610 non-redundant BESs that were presumed to derive from the nuclear genome. In total this non-redundant nuclear genome dataset surveys 35 Mb or ~4.3% of the pigeonpea genome.

A series of parallel analyses were performed to annotate the features of singletons and clustered BESs. Similarity to transcribed sequences or known proteins was assessed by BLAST'N' and BLAST'X' of sequences against the TIGR plant transcript assemblies http://plantta.jcvi.org/ and the National Center for Biotechnology Information (NCBI) non-redundant protein database, respectively, using an E-value cut-off of <1.00E^-20^. Further evidence of protein coding regions, as well as standardized nomenclature, was obtained by queries against the Interpro and GeneOntology Molecular Function databases. Similarity to known plant repeat sequences was assessed by BLAST'N' and tBLAST'X' against a database of plant repeat sequences (http://www.jcvi.org).

Based on the compiled information, BESs were subdivided into five primary categories: (1) non-annotated, (2) gene-containing, (3) retroelement-containing, (4) transposable element-containing, and (5) organelle- or ribosomal rRNA-containing, as shown in Table 1. Most sequence annotations were supported by multiple lines of evidence and a fraction of sequences were predicted to include both genes and either retroelements or transposable elements. Non-annotated sequences accounted for the majority of BAC ends, representing 53% of all non-redundant singletons and clusters, while nearly equal proportions of BESs were annotated as genes (21%) or retroelements (22%). It is likely that the retroelement category is an underestimate, because many of the most abundant Interpro descriptors within the "gene" category, such as "DNA/RNA Polymerase", are equally consistent with either "gene" or "retroelement". In the absence of additional annotation supporting classification as a retroelement, such sequences were classified as "gene".

**Table 1:** BAC-end sequence (BES) characteristics

Annotation	Retro-element (RE)	Genes (G)	G + RE	Transposable elements (TE)	G + TE	Non-annotated (NA)	Totals
**Total ends**	17928	17255	2,566	327	148	44,157	82,381
**Total unique clusters^a^**	7,401	11,233	913	201	44	32,147	51,939
**Average cluster depth**	2.42	1.54	2.81	1.63	3.36	1.37	1.69
**Total unique sequence^b^**	58,25,082	81,60,879	7,59,831	1,54,620	44,218	2,02,54,481	3,51,99,111
**Total clusters with SSRs**	302	1,083	13	11	0	3,188	4,597
**Total SSRs**	593	1,483	21	15	0	4100	6,212
**SSRs/100 Kbp**	10.2	18.2	2.8	9.7	0	20	17.7
**Selected SSR-BESs^d^**	124	646	4	2	0	1943	2,719
**Polymorphic SSRs^c^**	32	241	0	0	0	568	839
**Average number of alleles**	5.0 ± 1.7	5.4 ± 1.7	na	na	na	5.8 ± 2.1	5.7 ± 2.0
**Average PIC value**	0.53 ± 0.20	0.58 ± 0.18	na	na	na	0.57 ± .19	0.57 ± 0.18

^a^Total unique clusters represent the total number of sequence clusters plus the number of singleton (non-clustered) sequences.

^b^Total unique sequence represents the sum of the nucleotide length of all unique sequence clusters.

^c^Three polymorphic markers are from BAC ends annotated as "chloroplast" and are not listed in this table.

^d^2,964 SSR markers were derived from 2,719 BESs, with some BESs containing multiple SSRs

**Figure 2:**
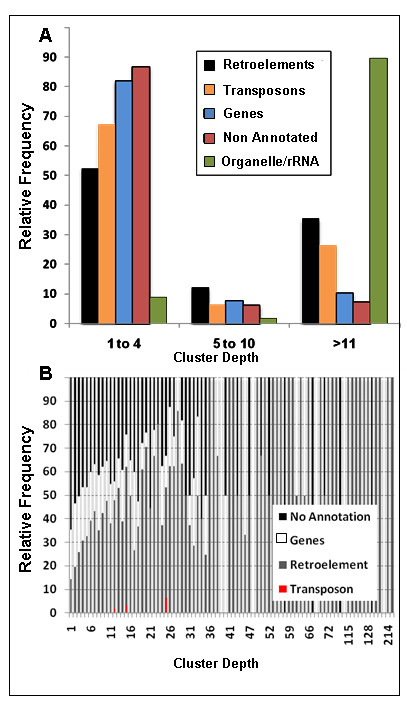
**Distribution of BAC end categories according to BES cluster depth**. Cluster depth supported the repetitive nature of mobile genetic elements while genic regions were mostly associated with less repetitive sequences.

Clustering of sequences as singletons or contigs provides a relative measure of sequence copy number (Table 1). As shown in Figure 2A and 2B, greater than 80% of sequences annotated as either gene or non-annotated were associated with clusters of depth <5 (Figure 2A) and their relative prevalence declined rapidly with cluster depth >1 (Figure 2B). By contrast, nearly 50% of all retroelement-containing sequences and 33% of all transponson-containing sequences were associated with clusters of depth >5, and they accounted for the vast majority of clusters with depth >10 sequences. Thus, sequence cluster depth supports the truism that mobile elements (i.e., retroelements and transposable elements) are often members of repetitive sequence families, while genes and intergenic regions (here we equate non-annotated sequences with intergenic regions) typically reside in less repetitive regions of the genome.

### Identification of BES-SSRs

With the goal of increasing genetic marker repertoire in pigeonpea, BESs (clusters + singletons) were surveyed for the presence of SSRs by means of the MIcroSAtellite (*MISA*) search module [19]. In total, 18,149 SSRs were identified, with mononucleotide (49% of total) and di-nucleotide (42% of total) repeats predominating. Excluding mono-nucleotide repeats, which were almost exclusively poly-A motifs, A/T-rich repeats accounted for 63% of all SSRs. The frequency of AT-rich repeats increased in rank order as motif length increased, from a low of 57% in di-nucleotide repeats to a high of 95% in penta-nucleotide repeats; this situation was absent only in the case of hexa-nucleotide repeats, where motifs with ≥50% GC content accounted for 53% of all repeats.

SSRs were either perfect SSRs (i.e., containing a single repeat motif such as 'TAA') or compound SSRs (i.e., composed of two or more SSRs separated by ≤100 bp). Perfect SSRs were further subdivided according to the length of SSR tracts [20]: Class I SSRs (≥ 20 nucleotides in length) and Class II SSRs (≥ 10 but < 20 nucleotides in length). Class I SSRs were enriched for di-nucleotide (69.2%) and tri-nucleotide repeats (17.2%), while Class II repeats were enriched in mono-nucleotide repeats (56.7%), with a less frequent occurrence of di- (37.1%) and tri-nucleotide (6.3%) repeats.

### Correlation between BAC end annotation and SSR occurrence

After excluding all mono-nucleotide repeat SSRs and SSRs with length <10 bp, the remaining 6,212 SSRs were selected for further analysis. These 6,212 SSRs were derived from 4,614 non-redundant BAC ends (singletons and clusters), 17 of which were annotated as organelle (15 chloroplast and 2 mitochondria).

**Figure 3:**
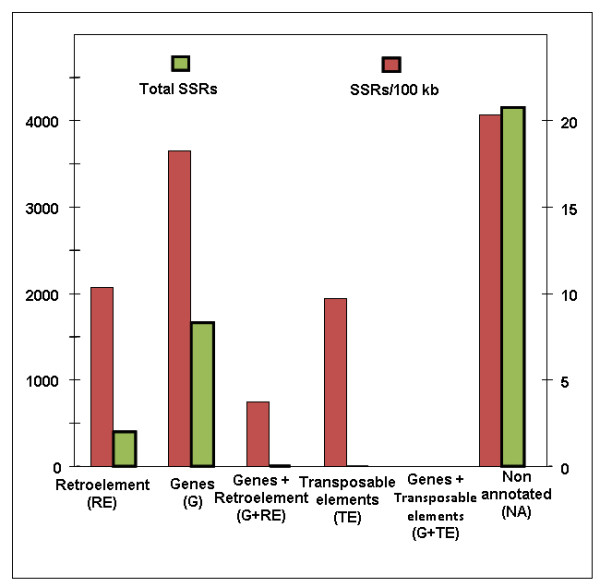
**Distribution and frequency of SSRs in differing genome fractions**. Maximum frequency and maximum amount of SSRs was exhibited by non annotated regions followed by the regions containing 'genes'.

The remaining 4,597 non-redundant BESs were divided among the four annotation categories, as shown in Table 1. Eighty-nine percent of these SSR-containing BESs (SSR-BESs) were either non-annotated or gene-containing, while 9.8% were retroelement-containing (Figure 3 and Table 1). The rate of SSR occurrence per 100 kb also differs considerably between annotation categories, consistent with the uneven discovery of SSRs between annotation categories. Thus, SSRs are twice as frequent per 100 kb in gene-containing (G) and non-annotated (NA) sequences compared to retroelement-containing (RE) sequences (Table 1 and Figure 3). Consistent with the likely pressure of purifying selection, BAC ends containing tri-nucleotide repeats were more likely to be annotated as genes (31%), compared to the remaining SSR-containing BAC sequences (22% annotated as genes).

For purposes of developing a uniform analysis of known pigeonpea SSRs, we obtained 457 SSRs submitted to NCBI GenBank by researchers at the University of Bonn (submitted by Odney *et al.*) and previously developed by our group (Varshney *et al.*). Both of these publicly available SSR sets were generated using PCR-based microsatellite enrichment strategies. As shown in the Table 1, the relative distribution of SSRs between genome fractions differs substantially for SSRs obtained by means of genome enrichment compared to random BAC end sequencing. In particular, genome-enrichment methodologies produced approximately three times the rate of retroelement-associated SSRs and an ~100-fold increase in the rate of SSRs derived from organelle or rRNA sequences, most of which were chloropast derived (data not shown).

### Development of novel SSR genetic markers

Primer pairs were designed and synthesized for a total of 3,072 non-redundant BAC-end sequence SSRs (BES-SSRs). We refer to these SSR markers as CcM (*Cajanus cajan *Microsatellite) (Additional file 1: List of newly developed SSR markers isolated from BESs of pigeonpea).

All 3,072 primer pairs were screened for amplification of DNA from two pigeonpea genotypes, i.e., ICP 28 and the popular variety "Asha", ICPL 87119. This analysis identified a set of 2,964 markers (96.5%) with scorable amplicons (Additional file 1: List of newly developed SSR markers isolated from BESs of pigeonpea). These 2,964 SSRs correspond to 2,719 BESs (Table 1), because some BESs contain multiple SSRs. Screening of these 2,964 markers on 22 pigeonpea genotypes, including 21 cultivated and one wild type (Table 2), further defined a subset of 842 polymorphic markers (28.4%). Among these polymorphic SSRs, allele count ranged from 2 to 14 (average of 5.65 alleles per marker) in the germplasm surveyed. 281 of the 842 polymorphic SSRs were polymorphic exclusively in wild species. Allelic data obtained from 22 genotypes were used to calculate the polymorphism information content (PIC) value of each CcM marker, and thus infer the discriminatory power of these CcM markers. PIC values ranged from 0.08 to 0.90 with an average of 0.57 (Additional file 2: Polymorphism status of SSR markers tested on 22 parental genotypes).

**Table 2:** List of genotypes used and their characters

Accession ID	Species	Maturity group	^c^DM	^d^DF	Seed color
ICP 28	*C. cajan*	-	-	-	-
ICPW 94	*C. scarabaeoides*	-	-	-	-
ICPB 2049	*C. cajan*	^a^MD	160	118	B
ICPL 99050	*C. cajan*	MD	175	123	B
ICPL 20096	*C. cajan*	MD	185	127	B
ICPL 332	*C. cajan*	MD	178	118	B
ICP 7035	*C. cajan*	MD	192	130	P
TTB7	*C. cajan*	-	-	-	-
ICPL 87091	*C. cajan*	^b^SD	121	74	C
ICPL 87119	*C. cajan*	MD	180	122	B
ICP 8863	*C. cajan*	MD	176	114	B
ICPL 20097	*C. cajan*	MD	187	131	B
ICPL 88034	*C. cajan*	SD	137	88	B
ICPL 84023	*C. cajan*	SD	134	68	B
ICPR 2671	*C. cajan*	MD	180	122	B
ICPA 2043	*C. cajan*	MD	175	115	B
ICPR 3467	*C. cajan*	-	-	-	-
ICPR 2438	*C. cajan*	-	-	-	-
ICPA 2039	*C. cajan*	MD	122	80	
ICPR 2447	*C. cajan*	-	-	-	-
ICPL 20108	*C. cajan*	MD	181	125	C
ICP 2376	*C. cajan*	MD	176	118	C

^a^MD: medium duration, ^b^SD: short duration, ^c^DM: days to maturation, ^d^DF: days to flowering, B: brown, P: purple, C: cream

As shown in Table 3, Class I SSRs were on average more polymorphic (328 of 900, or 36.4%) than Class II SSRs (287 of 1,438, or 20.0%), with mean PIC values of 0.60 and 0.53 (significant at p < 0.0001), respectively. Within this set of perfect SSRs, di-nucleotide repeats accounted for the largest number of polymorphic loci i.e. 39.9% for Class I and 22.8% for Class II). SSRs derived from compound repeats had an average polymorphism rate of 36.3%, similar to Class I SSRs. The average genotype pair was distinguished by 137 polymorphic SSRs (Table 4). As expected, however, polymorphism rates varied considerably depending on the genotype pair under comparison, from a low of 52 polymorphic SSRs (ICPL 332 × ICPL 20096) to a high of 378 polymorphic SSRs (ICP 28 × ICPW 94).

**Table 3:** Distribution of polymorphic markers into different repeat classes

SSR type	Repeat classes	Number of markers synthesized	Number of markers amplified	Number of polymorphic markers	PIC value	Number of alleles
**Compound**		657	626 (95.28%)	227 (36.26%)	0.08-0.88(0.58)	2-12 (5.74)
						
**Perfect**						
***Class I***						
	NN	639	592 (92.64%)	236 (39.86%)	0.08-0.90 (0.60)	2-14 (6.55)
	NNN	200	194 (97%)	66 (34.02%)	0.08-0.85 (0.60)	2-13 (5.87)
	NNNN	62	61(98.38%)	14 (22.95%)	0.28-0.81 (0.50)	3-9 (4.71)
	NNNNN	10	10 (100%)	2 (20%)	0.52-0.76 (0.64)	5-7 (6)
	NNNNNN	43	43 (100%)	10 (23.25%)	0.52-0.76 (0.64)	2-7 (4.4)
	Total	954	900 (94.33%)	328 (36.44%)		
***Class II***						
	NN	1,006	987 (98.11%)	219 (22.18%)	0.08-0.83 (0.53)	2-9 (4.9)
	NNN	455	451 (99.12%)	68 (15.07%)	0.08-0.74 (0.48)	2-6 (4.4)
	Total	1,461	1,438 (98.42%)	287 (19.95%)		
	Grand Total	3,072	2,964 (96.48%)	842 (28.40%)		

**Table 4:** SSR polymorphism status on 13 mapping populations

Mapping parents	^**a**^**Segregating ** traits	Number of ** F**_**2 **_**lines**	Number of polymorphic markers
ICP 28 × ICPW 94	PB	79	378
ICPB 2049 × ICPL 99050	FW	370	103
ICP 332 × ICPL 7035	SMD	-	84
ICPL 332 × ICPL 20096	FW and SMD	384	52
ICPL 87119 × ICPL 87091	FW and SMD	124	114
ICPL 8863 × ICPL 20097	SMD	384	143
ICPL 88034 × ICPL 84023	WL	-	106
ICPA 2043 × ICPR 2671	FR	243	179
ICPA 2043 × ICPR 3467	FR	261	173
ICPA 2039 × ICPR 2447	FR	123	149
ICPA 2039 × ICPR 2438	FR	240	137
ICPL 20102 × ICP 2376	FW	-	84
TTB7 × ICP 7035	SMD	144	80

^a^PB: pod borer, FW: *Fusarium *wilt, SMD: sterility mosaic disease, WL: water logging,

FR: fertility restoration

### Construction of an SSR-based genetic map

An inter-specific F_2 _population derived from ICP 28 (*C. cajan*) × ICPW 94 (*C. scaraboides*) was selected for the construction of a reference genetic map. Consistent with a wide genetic cross, this pairwise comparison had the highest number of polymorphic SSRs (Table 4). The mapping population was genotyped with all polymorphic markers and marker segregation data were analyzed by the goodness of fit test for a 1:2:1 segregation ratio. Only 138 (36.50%) markers showed good agreement with the expected segregation ratio 1:2:1 (at the threshold of *p *= 0.05). Among the 240 markers with deviation from Mendelian ratios we observed instances of complete absence or very low occurence of one parental allele, and instances of excess heterozygosity.

**Figure 4:**
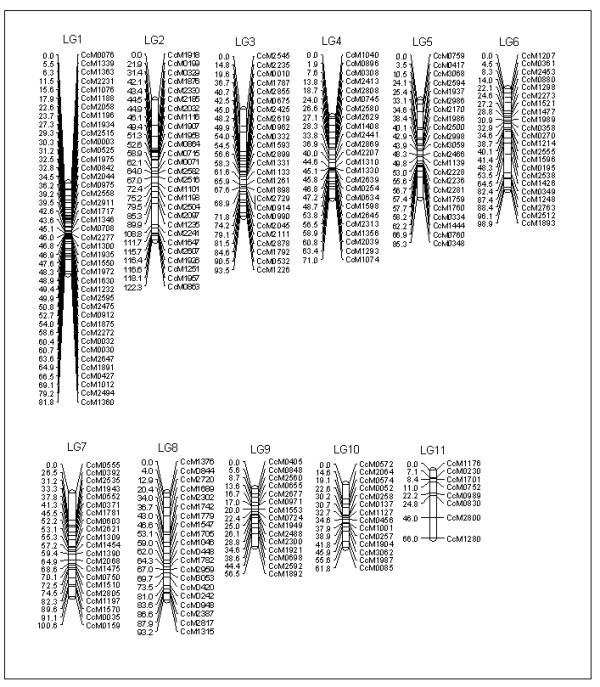
**Reference genetic map of pigeonpea derived from an inter-specific F_2 _population (ICP 28 × ICPW 94)**. Initially, a skeleton map with normally segregating markers was constructed using MAPMAKER/EXP 3.0 while further integration of additional markers was performed with Joinmap 3.0 by keeping the mapmaker order as "fixed". Distances between the loci (in cM) are shown to the *left *of the linkage group and all the *loci *at the right side of the map.

The genetic linkage map was constructed in a stepwise manner, beginning with the 138 normally segregating markers at LOD 5 and a minimum recombination fraction of 37.5. Subsequently, the 240 distorted markers were tested for integration with the help of Joinmap 3.0 software. The combined 239 markers yielded a genetic map of 930.90 cM (919 kb/cM) (Figure 4), with an average of 21 markers per linkage groups and an average between marker distance of 3.8 cM. A total of 11 linkage group could be assigned, and these are presumed to correspond to the haploid chromosome set of *C. cajan *(n = 11).

### Identification of informative SSR markers for hybrid purity assessment

In pigeonpea, there is a need for genetic markers to assess hybrid seed purity. Among the genotypes surveyed for SSR polymorphism (Table 4), four genotypes (ICPA 2039, ICPR 2438, ICPA 2043 and ICPR 2671) have been used for the development of two hybrids: ICPH 2438 (ICPA 2039 × ICPR 2438) and ICPH 2671 (ICPA 2043 × ICPR 2671) [5,21]. For each hybrid, 42 polymorphic markers were selected that distinguished the parental lines and which gave high quality amplification in prior analyses. To assess the reliability of these SSR markers, 183 seeds of ICPH 2438 and 174 seeds of ICPH 2671 were obtained from the ICRISAT germplasm and analyzed together with seeds of parental lines. Based on this analysis, both ICPH 2438 and ICPH 2671 seed stocks had high rates of purity (96.3% and 94.8%, respectively). However, the frequency with which tested hybrids showed banding patterns typical of both parental alleles was dependent upon the markers under analysis. Accordingly the marker wise hybrid purity index varied between markers, ranging from 31.88% (CcM0724) to 99.42% (CcM0752) for ICPH 2671 and from 71.26% (CcM0133) to 100% (CcM2241) for ICPH 2438. A total of 30 markers for ICPH 2671 and 35 markers for ICPH 2438 could detect purity between 90 - 100% (Additional file 3: Purity index of polymorphic SSR markers on pigeonpea hybrid ICPH 2671 individuals and Additional file 4: Purity index of polymorphic SSR markers on pigeonpea hybrid ICPH 2438 individuals). The frequency of heterozygosity for the hybrid in ICPH 2438 ranged from a minimum of 53.1% (23/42) to a maximum of 100% (42/42). In case of ICPH 2671 heterozygosity for a hybrid ranged from minimum 53.1% (23/42) to a maximum of 95.24% (40/42).

**Figure 5:**
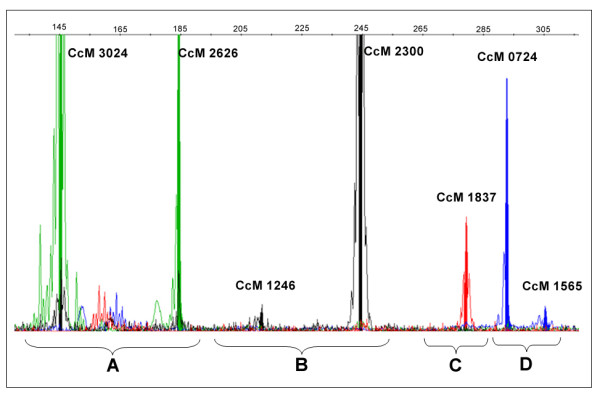
**Electropherogram display for the multiplex set MG 1 for purity assessment of hybrid ICPH 2671**. This figure shows the analysis (GENEMAPPER output) of seven SSR markers of MG1 for ICPH 2671 in a single capillary. SSR markers labeled with the same fluorescence dye are analyzed in individual panels. A. Analysis of two VIC (green) labeled SSR markers, B. Two NED (black) labeled SSR markers, C. One PET (red) labeled SSR markers, and D. Analysis of two FAM (blue) labeled SSR markers.

With the objective of reducing the cost and time of PCR assays for purity assessment, we identified sets of SSRs with allele sizes that were sufficiently different to permit multiplex analysis of hybrid seeds. In the case of ICPH 2671, 35 of the 42 markers were assigned to 9 multiplex groups (MG 1- MG 9, Table 5). Figure 5 shows the example of multiplexing the 7 ICPH 2671 MG 1 markers. Similarly for ICPH 2438, 26 of the 42 markers were assigned to 12 marker groups. A single multiplex of four markers (CcM0257, CcM1559, CcM1825 and CcM1895) produced well resolved polymorphisms on both ICPH 2671 and ICPH 2438.

**Table 5:** Details on marker groups (MGs) for multiplex assays for assessing purity of two hybrids

Hybrid/Marker group ID	Number of markers	Marker names
**ICPH 2671**^a^		
MG 1	7	CcM0724:CcM2626:CcM2300:CcM1837:CcM1565:CcM3024:CcM1246
MG 2	7	CcM2350:CcM0737:CcM2517:CcM1825:CcM2228:CcM0516:CcM0171
MG 3	6	CcM2802:CcM2704:CcM2097:CcM0021:CcM1459:CcM0752
MG 4	6	CcM1895:CcM2076:CcM2948:CcM1707:CcM2257:CcM2281
MG 5	5	CcM1232:CcM1053:CcM1139:CcM2401:CcM0207
MG 6	4	CcM2370:CcM0374:CcM0257:CcM0246
MG 7	2	CcM1984:CcM0252
MG 8	2	CcM1385:CcM2453
MG 9	2	CcM1559:CcM0948
		
**ICPH 2438**		
MG 1	8	CcM1825:CcM0878:CcM2672:CcM0057:CcM1713:CcM1651:CcM1647:CcM2330
MG 2	6	CcM1338:CcM1669:CcM2492:CcM2413:CcM0858:CcM1251
MG 3	5	CcM0121:CcM0008:CcM0257:CcM2380:CcM1371
MG 4	5	CcM1559:CcM2241:CcM1895:CcM0402:CcM1406
MG 5	4	CcM2386:CcM2449:CcM1565:CcM0207
MG 6	2	CcM0522:CcM1438
MG 7	2	CcM2164:CcM2781
MG 8	2	CcM0133:CcM0195
MG 9	2	CcM0481:CcM2595
MG10	2	CcM0948:CcM1282
MG11	2	CcM1616:CcM0252
MG12	2	CcM2982:CcM1078

^a^Out of 42 informative markers identified for ICPH 2671, only 41 primers could be grouped in different multiplexes and the marker CcM1277 could not be multiplexed in any marker group.

## Discussion

The narrow genetic base of pigeonpea has hindered the wide use of molecular marker technology for crop improvement [22]. In the present study, two BAC libraries were developed with an estimated ~11× genome coverage of pigeonpea. Sequencing of 50,000 BAC clones from both insert ends provided 88,860 BESs. Removal of cytoplasmic orgeneller BESs and cluster analysis facilitated the maximum possible recovery of nuclear genomic sequences comprising 41,329 singletons and 10,601 non-redundant contigs. With an objective to understand the constitution of SSR containing BAC clones, BESs were run through an annotation pipeline. Major proportion of the sequences remained non-annotated which may be considered as 'novel' *C*. *cajan *sequences. The overall repetitive fraction, resulting from BES analysis was found to be intermediate (22.15%) when compared with the percentage of repetitive elements in BESs of other legumes such as *Trifolium *(8.5%), soybean (33.5%), and common bean (49.3%) [23]. BES annotation analysis has shown a considerable variability in the amount of repetitive fraction in different crop species such as tomato (49.3%) [24], papaya (16%) [25], banana (36%) [26] and citrus (25%) [27]. This variation in the amount of repetitive elements in BESs is an indicative feature of presence of repetitive elements in the genome of a species. A varying level of annotations in different species may also be responsible for difference in repetitive elements. Proportion of annotated genic fraction was found more or less similar as observed in the BESs analysis of other crop species such as *Phaseolus *(29.3%) [23], apple (10.9%) [28], banana (11%) [26], *Brassica *(11%) [29] and papaya (19.%) [25].

BESs have been very useful to develop SSR markers in several plant species including legumes like soybean [17], common bean [23] and *Medicago *[16]. In terms of SSRs abundance, overall density of 1 SSR per 5.64 kb seems to be in good congruency with the earlier reports in plant genomes [30]. Similar results showing SSR frequencies of 1 SSR per 4 to 10 kb were achieved in different plant species like *Medicago*, soybean, *Lotus*, *Arabidopsis *and rice [16]. This discrepancy observed in different studies may be accounted to (i) amount of sequence data analyzed, (ii) criteria for SSR identification, and (iii) different sources of derived sequences. It is also important to note that after excluding non-annotated BESs, majority (70.21%) of SSRs belong to be associated with genes. These observations are in agreement of the comprehensive study in plant genomes where SSRs were found associated mainly with genes [31].

In terms of distribution of SSRs, unlike the common occurrence of 'CG' motif in monocot species, 'CG' motifs were the least abundant in pigeonpea genome, as previously observed in other legume species (*Medicago*, *Lotus *and soybean). Such low abundance of "CG" di-nucleotide repeats may be attributed to their tendency of forming secondary structures (hairpins), leading to a selective pressure against 'CG' accumulation in genomes [32].

While converting identified SSRs into genetic markers, though 3,072 SSR primer pairs were synthesized; of these 2,964 (96.48%) primers yielded scorable amplicons. This rate of successful amplification is quite higher than earlier reported in pigeonpea [10-13]. All the repeat classes showed more than 98% amplification except di-nucleotide repeats which had comparatively lower rate of amplification (95.98%).

All the successfully amplified primer pairs were screened for polymorphism on a set of 22 diverse pigeonpea genotypes representing parents of 13 mapping populations segregating for various traits. These mapping populations represented the best cross combinations based on diversity revealed through morphological attributes and available marker data [33]. The overall frequency of length polymorphism was found to be 28.40% which is lower than reported in earlier studies i.e. 50% [10], 81.3% [13] and 95% [11]. This can be attributed to use of only one wild species genotype in this study unlike earlier studies. Occurrence of a very low level of DNA polymorphism among pigeonpea cultivars is not unexpected as several studies have documented such results [33-35].

As expected degree of marker polymorphism was lower in intra-specific populations than in inter-specific mapping population (ICP 28 × ICPW 94). The frequency of marker polymorphism increased dramatically with SSR locus longer than 200 bp. PIC values for SSR markers were also analyzed in relation to repeat length and unit type. In terms of repeat length, Class I SSRs were more polymorphic as compared to the Class II SSRs which may be accounted to the hyper-variable nature of Class I SSRs [20] Among different type of repeat unit classes, tetra-nucleotide repeats, in general, showed the higher average PIC value (0.64) followed by di-nucleotide repeats (0.57). It was also observed that among tri-nucleotide repeat class, the 'TAA' repeat motifs, displayed higher polymorphism (average PIC value = 0.59). Similarly, 'TA' repeat motifs in di-nucleotide repeat class had a higher average PIC value (0.59) compared to the others. Similar trends were also observed in other legumes such as chickpea [36], [16] and [37] where the SSR markers with repeat motifs 'TAA' or 'TA' exhibited extensive abundance and polymorphism as well. Higher average PIC value of compound SSRs (0.58) can be attributed to the fact that the markers with compound SSRs have more than one SSR motif, which increases their chance to be polymorphic [9].

This study provides a list of polymorphic markers for different mapping populations that segregate for a number of important traits like *Fusarium *wilt (FW), sterlity mosaic disease (SMD), fertility restorer (*Rf*) etc. that are important for pigeonpea improvement [38]. Genotyping of these mapping populations with identified polymorphic markers together with phenotyping data should provide the markers associated with QTLs (quantitative trait loci)/gene(s) for trait of interest that can be used for enhancing the breeding efficiency through marker-assisted selection.

To develop a reference genetic map, an inter-specific cross was used so that a larger number of segregating loci can be integrated into the genetic map. Usually SSR markers are co-dominant and follow Mendelian inheritance [39]. However deviation from the expected segregation ratio for SSR markers is not an uncommon feature in inter-specific crosses and especially F_2 _population. Significant distortion observed in the marker data may be attributed to several possible reasons such as the abortion of male or female gametes or the selective exclusion of a particular gametic genotype from fertilization, owing to incompatibility, incongruity, certation, or zygote selection [40]. Percentage distortion observed in the present study is comparable with previously reported studies performed on inter-specific crosses [41].

In the present study, the genetic map derived from an inter-specific cross ICP 28 × ICPW 94 included eleven discrete linkage groups corresponding to the basic chromosome number of the genus (x = 11). Initial construction of a skeletal map with un-skewed markers and followed by integration of distorted markers helped in minimizing the possibility for spurious assignments of markers [42]. The final map comprised of 239 marker loci with a total map length of 930.90 cM having average spacing of 3.8 cM between two marker loci. This is the first report on the construction of SSR-based genetic map in pigeonpea. Therefore this map should serve as a 'reference map' for other future genetic maps of pigeonpea. Moreover as the SSR markers are derived from the BAC-end sequences, these markers and the map should be very useful resource for linking the genetic map with a 'future' physical map of pigeonpea [38].

Developed set of large number of SSR markers should be very useful for applied aspects of genetics and breeding in pigeonpea, especially when the cultivated gene pool has a narrow genetic diversity. In case of pigeonpea, CMS- hybrid technology is becoming popular to tackle the low crop productivity [5]. For assessing the genetic purity of hybrids, in general, grow out test (GOT) based on morphological criteria is used. However, GOT is limited by the accuracy, time and labour cost [43]. In this context, for each of two hybrids (ICPH 2671 and ICPH 2438), a set of 42 markers has been identified that can be used for purity assessment of hybrid seeds. SSR markers have been found very effective for determining hybrid purity in many species like rice [44], maize [45] and cotton [46]. In fact in case of ICPH 2438 hybrid, two diagnostic SSR markers were identified for purity assessment in an earlier study also [21]. Although some studies report suitability of even one marker for hybrid purity assessment test [43,47,48]. This study increases the diagnostic markers in large number for ICPH 2438 and also identifies a set of diagnostic markers for another pigeonpea hybrid ICPH 2671. Moreover identification of different marker groups, especially the group of common markers (CcM0257, CcM1559, CcM1825 and CcM1895) for both hybrids, for undertaking multiplex assays provides an added value to enhance their utility for hybrid purity assessment.

## Conclusion

In summary this study reports a large-scale development of SSR markers and construction of SSR based genetic map in pigeonpea for the first time. In addition, a large number of informative SSR markers that can be used in multiplexes for assessing the seed purity of two hybrids. It is anticipated that SSR markers and the genetic map reported in this study should provide a reference resource for construction and comparison of genetic maps for new mapping populations, finger printing and cultivar identification, assessment of genetic diversity and gene flow among *Cajanus *species. New genetic maps, to be devloped based on polymorphic markers identified in this study, will facilitate trait mapping and marker assisted selection. Furthermore, genomic SSR markers identified from BESs and integrated into genetic maps provide a valuable resource for anchoring future physical map or whole genome sequence to the genetic map.

## Methods

### Plant material and DNA extraction

Two pigeonpea genotypes namely ICP 28 and ICPL 87119 ("Asha") were employed for checking the amplification of SSR loci with newly designed primer pairs. To identify informative set of SSR markers, a set of 22 genotypes was utilized for screening the polymorphism (Table 2). These genotypes represent parents of 13 mapping populations which are segregating for various agronomical important traits.

A F_2 _population of 79 individuals derived from an inter-specific cross of ICP 28 (*Cajanus cajan *accession) and ICPW 94 (*Cajanus scarabaeoides *accession) was used for development of a genetic map.

For assessment of genetic purity of hybrids ICPH 2438 and ICPH 2671, a set of 183 and 174 seeds of two cytoplasmic-nuclear male-sterility (CMS) based hybrids (obtained from ICRISAT) were used respectively. Total genomic DNA from leaf tissue was isolated and purified according to protocol provided by Cuc and colleagues [49].

### BAC-end sequence (BES) data

Two BAC libraries were constructed by using *Hin*dIII and *Bam*HI restriction enzymes. The *Hin*dIII library was composed of 34,560 clones with an estimated average insert size of 120,000 bp, while the *Bam*HI library was composed of 34,560 clones with an estimated average insert size of 115,000 bp. These clones collectively represented ~11× coverage of the pigeonpea genome. A total of 50,000 BAC clones were attempted for end-sequencing. BAC clones were inoculated into Luria Broth (LB) media containing appropriate antibiotic (chloramphenicol or kanamycin) and incubated in a shaking incubator. BAC-DNA was purified by alkaline lysis solutions. Big dye terminator chemistry was used to end sequence the BAC clones. Post reaction removal of excess dye was performed using a Sephadex G50 mini-column filter plate method. Sequences were analyzed with an automated sequencer. Base calling and sequence trimming were performed with PHRED software [50]. The PHRED output was converted into FASTA format and vector sequences were masked. Terminal vector sequences were then trimmed and BESs shorter than 100 bp were discarded and the remaining 88,860 BESs were then used for mining of SSRs.

### Mining of SSRs

BESs were used for mining the SSRs using Perl based *MI*cro*SA*tellite (*MISA*) http://pgrc.ipk-gatersleben.de/misa[19] search module which is capable of identifying perfect as well as compound SSRs. All BESs with a minimum size of 100 bp were arranged in a single text file in FASTA format and this file was used as an input for *MISA*. The criteria used for the identification of true SSRs included minimum ten repeats for mono (N)-, six repeats for di (NN)- and five repeats for tri (NNN)-, tetra (NNNN)-, penta (NNNNN)- and hexa (NNNNNN)- nucleotide repeat units. Two SSRs separated by maximum 100 nucleotide bases were considered as part of a compound SSR. Sequence complementarity was considered while classifying identified SSRs under different classes.

### Primer designing

For generating the genetic markers, redundancy in the identified SSRs from BESs was taken into account. Cluster analysis was done on the BESs to identify non-redundant sequences. In general, one SSR containing BES was selected from each cluster for designing the primer pairs.

Designing of primer pairs for identified SSRs was done by using standalone Primer3 http://frodo.wi.mit.edu/ program using *MISA *generated Primer3 input file [19]. The criteria used for designing primer pairs included annealing tempeature (T_m_) range of 57°C - 60°C with an average of 59°C, amplicon size 100 - 280 bp, primer length 20 ± 5 bp and GC% 50 ± 5. M13 dye labeled primer pairs were synthesized for the selected SSRs.

### Amplification and separation of SSR loci

Polymerase chain reactions (PCRs) for amplification of SSR loci were performed in a 5 μl reaction volume [0.5 μl of 10× PCR buffer, 1.0 μl of 15 mM MgCl_2_, 0.25 μl of 2 mM dNTPs, 0.50 μl of 2 pM/μl primer anchored with M13-tail (MWG-Biotech AG, Bangalore, India), 0.1 U of *Taq *polymerase (Bioline, London, UK), and 1.0 μl (5 ng/μl) of template DNA] in 96-well micro titre plate (ABgene, Rockford, IL, USA) using thermal cycler GeneAmp PCR System 9700 (Applied Biosystems, Foster City, CA, USA). A touch down PCR programme was used to amplify the DNA fragments: initial denaturation was for 5 min at 95°C followed by 5 cycles of denaturation for 20 sec at 94°C, annealing for 20 sec at 60°C (the annealing temperature for each cycle being reduced by 1°C per cycle) and extension for 30 sec at 72°C. Subsequently, 35 cycles of denaturation at 94°C for 20 sec followed by annealing for 20 sec at 56°C and extension for 30 sec at 72°C and 20 min of final extension at 72°C. PCR products were checked for amplification on 1.2% agarose gel. Separation of amplified products on capillary electrophoresis using GeneMapper software version 4.0 (Applied Biosystems, Foster City, CA, USA) was undertaken.

### Polymorphism information content (PIC)

PIC value of all polymorphic SSR markers was calculated as follows [51]

where k is the total number of alleles detected for a given marker locus and *Pi *is the frequency of the i^th ^allele in the set of genotypes investigated.

### Linkage mapping

Segregation data obtained for polymorphic SSR markers on the F_2 _population were used for linkage mapping. Due to segregation distortion for some SSR loci, initially a framework genetic map was prepared with normally segregating markers at logarithm of odds (LOD) of 5 with a minimum recombination threshold of 37.5 using MAPMAKER/EXP 3.0 [52]. Initially 'Group' command was used to group markers in various linkage groups. Then 'Compare' and 'Try' commands were used to locate the SSR markers within each linkage group. The ordered marker sequences were confirmed by the 'Ripple' command and finally the linkage groups were generated by 'Map' command. Kosambi mapping function was used to convert recombination frequency into map distances [53]. The whole data set was then analyzed with the help of JoinMap 3.0 software [54]. Linkage groups were established at LOD ≥ 3 with other parameters like recombination threshold of 0.40, ripple value of 1 and jump threshold of 5. The framework map order was fixed as 'anchor' using 'fixed order' command and all the remaining markers including the distorted ones were integrated because with JoinMap, the risk of errors in the placement of distorted markers to a linkage group are minimized [55]. Final linkage maps were drawn with the help of Mapchart version 2.2 [56].

### Hybrid purity assessment

DNA extraction and PCR amplification of each seed of hybrids was done as described previously. SSR allele data for the hybrid seeds was recorded as "A" [allele of male- sterile parent (A- line)], "B" [allele of fertility restorer parent (R- line)] and "H" (alleles from both the parents "Hybrid") format. Purity index for each marker was calculated using scored data by applying the following formula:

## Abbreviations

BAC: Bacterial artificial chromosome; BESs: BAC-end sequences; SSRs: Simple sequence repeats; PCRs: Polymerase chain reactions; PIC: Polymorphism information content; QTLs: Quantitative trait loci; CMS: Cytoplasmic-nuclear male-sterility

## Authors' contributions

AB and AD conducted SSR genetic mapping experiments, analyzed data and participated in preparing the first draft of the manuscript; GS and NK participated in marker polymorphism experiments, RK, KN, KBS, SR were engaged in hybrid purity testing experiments; RVP, ADF, CDT, GDM, DRC and RKV contributed to construction of BAC-libraries, sequencing the BAC-ends and BES anaysis; HDU generated the mapping population; RG, DS, PBK, NKS, HDU, CDT, GDM together with DRC and RKV participated in data analysis and interepreting the results; RKV and DRC conceived this study, planned experiments and, together with AB and AD, finalized the manuscript. All authors have read the manuscript.

## Supplementary Material

Additional file 1 **List of newly developed SSR markers isolated from BESs of pigeonpea**. List of newly developed BES-SSRs providing details on corresponding GenBank ID, SSR motif, primer sequences, product size and amplification status. Click here for file

Additional file 2 **Polymorphism status of SSR markers tested on 22 parental genotypes**. Detailed information on markers, exhibiting polymorphism in at least one parental combination, along with their SSR motifs, number of alleles and PIC values. Click here for file

Additional file 3 **Purity index of polymorphic SSR markers on pigeonpea hybrid ICPH 2671 individuals**. List of polymorphic markers between parental lines (ICPA 2043 and ICPR 2671) and corresponding purity percentage of designated hybrid. Click here for file

Additional file 4 **Purity index of polymorphic SSR markers on pigeonpea hybrid ICPH 2438 individuals**. List of polymorphic markers between hybrid parents (ICPA 2039 and ICPR 2438) and percentage of purity assessed by these markers in designated hybrids. Click here for file

## References

[B1] Greilhuber J Obermayer R Genome size variation in *Cajanus cajan *(Fabaceae): a reconsideration Plant Syst Evol 1998 212 135 141 10.1007/BF00985225

[B2] van der Maesen LJG Nene YL, Hall SD, Sheila VK Pigeonpea: origin, history, evolution and taxonomy Pigeonpea 1990 Wallingford: CAB International 15 46

[B3] Reddy LJ Faris DG A cytoplasmic male sterile line in pigeonpea International Pigeonpea Newslett 1981 1 16 17

[B4] Marley PS Hillocks RJ Effect of root-knot nematodes (*Meloidogyne *spp.) on *Fusarium *wilt in pigeonpea (*Cajanus cajan*) Field Crop Res 1996 46 15 20 10.1016/0378-4290(95)00083-6

[B5] Saxena KB Sultana R Mallikarjuna N Saxena RK Kumar RV Sawargaonkar SL Varshney RK Male-sterility systems in pigeonpea and their role in enhancing yield Plant Breed 2010 129 125 134 10.1111/j.1439-0523.2009.01752.x

[B6] Varshney RK Hoisington DA Tyagi AK Advences in cereal genomics and applications in crop breeding Trends Biotechnol 2006 24 490 499 10.1016/j.tibtech.2006.08.006 16956681

[B7] Varshney RK Thudi M May GD Jackson SA Legume genomics and breeding Plant Breed Rev 2010 33 257 304

[B8] Jones N Ougham H Thomas H Pasakinskiene I Markers and mapping revisited: finding your gene New Phytol 2009 183 935 966 10.1111/j.1469-8137.2009.02933.x 19594696

[B9] Gupta PK Varshney RK The development and use of microsatellite markers for genetic analysis and plant breeding with emphasis on bread wheat Euphytica 2000 113 163 185 10.1023/A:1003910819967

[B10] Burns MJ Edwards KJ Newbury HJ Ford-Lloyd BR Baggot CD Development of simple sequence repeat (SSR) markers for the assessment of gene flow and genetic diversity in pigeonpea (*Cajanus cajan*) Mol Ecol Notes 2001 1 283 285 10.1046/j.1471-8278.2001.00109.x

[B11] Odeny DA Jayashree B Ferguson M Hoisington D Cry LJ Gebhardt C Development, characterization and utilization of microsatellite markers in pigeonpea Plant Breed 2007 126 130 136 10.1111/j.1439-0523.2007.01324.x

[B12] Odeny DA Jayashree B Gebhardt C Crouch J New microsatellite markers for pigeonpea (*Cajanus cajan *(L.) Millsp.) BMC Research Notes 2009 2 35 10.1186/1756-0500-2-35 19284532 PMC2660351

[B13] Saxena RK Prathima C Saxena KB Hoisington DA Singh NK Varshney RK Novel SSR markers for polymorphism detection in pigeonpea (*Cajanus *spp.) Plant Breed 2010 129 142 148 10.1111/j.1439-0523.2009.01680.x

[B14] Varshney RK Graner A Sorrells ME Genic microsatellite markers in plants: features and applications Trends Biotechnol 2005 23 48 55 10.1016/j.tibtech.2004.11.005 15629858

[B15] Varshney RK Nayak SN May GD Jackson SA Next generation sequencing technologies and their implications for crop genetics and breeding Trends Biotechnol 2009 27 522 530 10.1016/j.tibtech.2009.05.006 19679362

[B16] Mun JH Kim DJ Choi HK Gish J Debelle F Mudge J Denny R Endre G Saurat O Dudez AM Kiss GB Roe B Young ND Cook D Distribution of microsatellites in the genome of *Medicago truncatula*: a resource of genetic markers that integrate genetic and physical maps Genetics 2006 172 2541 2555 10.1534/genetics.105.054791 16489220 PMC1456377

[B17] Shultz JL Samreen K Rabia B Jawaad AA Lightfoot DA The development of BAC-end sequence-based microsatellite markers and placement in the physical and genetic maps of soybean Theor Appl Genet 2007 114 1081 1090 10.1007/s00122-007-0501-9 17287974

[B18] Schlueter JA Lin JY Schlueter SD Vasylenko SIF Deshpande S Yi J O'Bleness M Roe BA Nelson RT Scheffler BE Jackson SA Shoemaker RC Gene duplication and paleopolyploidy in soybean and the implications for whole genome sequencing BMC Genomics 2007 8 330 10.1186/1471-2164-8-330 17880721 PMC2077340

[B19] Varshney RK Thiel T Stein N Langridge P Graner A *In silico *analysis on frequency and distribution of microsatellites in ESTs of some cereal species Cell Mol Biol Lett 2002 7 537 546 12378259

[B20] Temnykh S DeClerck G Lukashova A Lipovich L Cartinhour S McCouch S Computational and experimental analysis of microsatellites in rice (*Oryza sativa *L.): frequency, length variation, transposon associations, and genetic marker potential Genome Res 2001 11 1441 1452 10.1101/gr.184001 11483586 PMC311097

[B21] Saxena RK Saxena KB Varshney RK Application of SSR markers for molecular characterization of hybrid parents and purity assessment of ICPH 2438 hybrid of pigeonpea [*Cajanus cajan *(L.) Millspaugh] Mol Breed 2010 26 371 380 10.1007/s11032-010-9459-4

[B22] Saxena KB Genetic improvement of pigeonpea-a review Trop Plant Biol 2008 1 159 178 10.1007/s12042-008-9014-1

[B23] Schlueter JA Goicoechea JL Collura K Gill N Lin JY Yu Y Vallejos E Munoz M Blair MW Tohme J Tomkins J McClean P Wing R Jackson SA BAC-end sequence analysis and a draft physical map of the common bean (*Phaseolus vulgaris *L.) genome Trop Plant Biol 2008 1 40 48 10.1007/s12042-007-9003-9

[B24] Budiman MA Mao L Wood TC Wing RA A deep coverage tomato BAC library and prospects toward development of an STC framework for genome sequencing Genome Res 2000 10 129 136 10645957 PMC310507

[B25] Lai CW Yu Q Hou S Skelton RL Jones MR Lewis KL Murray J Eustice M Guan P Agbayani R Moore PH Ming R Presting GG Analysis of papaya BAC end sequences reveals first insights into the organization of a fruit tree genome Mol Genet Genomics 2006 276 1 12 10.1007/s00438-006-0122-z 16703363

[B26] Cheung F Town CD A BAC end view of the *Musa acuminata *genome BMC Plant Biol 2007 7 29 10.1186/1471-2229-7-29 17562019 PMC1904220

[B27] Terol JM Naranjo A Ollitrault P Talon M Development of genomic resources for *Citrus clementina*: characterization of three deep-coverage BAC libraries and analysis of 46,000 BAC end sequences BMC Genomics 2008 9 423 10.1186/1471-2164-9-423 18801166 PMC2561056

[B28] Han Y Korban SS An overview of the apple genome through BAC end sequence analysis Plant Mol Biol 2008 67 581 588 10.1007/s11103-008-9321-9 18521706

[B29] Hong CP Piao ZY Kang TW Batley J Yang TJ Hur YK Bhak J Park BS Edwards D Lim YP Genomic distribution of simple sequence repeats in *Brassica rapa* Mol Cells 2007 23 3 49 356 17646709

[B30] Cardle L Ramsay L Milbourne D Macaulay M Marshall D Waugh R Characterization of physically clustered simple sequence repeats in plants Genetics 2000 156 847 854 11014830 10.1093/genetics/156.2.847 PMC1461288

[B31] Morgante M Hanafey M Powell W Microsatellites are preferentially associated with nonrepetitive DNA in plant genomes Nat Genet 2002 30 194 200 10.1038/ng822 11799393

[B32] Eustice M Yu Q Lai CW Hou S Thimmapuram J Liu L Alam M Moore PH Presting GG Ming R Development and application of microsatellite markers for genomic analysis of papaya Tree Genet Genomes 2008 4 333 341 10.1007/s11295-007-0112-2

[B33] Saxena RK Saxena KB Kumar RV Hoisington DA Varshney RK Simple sequence repeat-based diversity in elite pigeonpea genotypes for developing mapping populations to map resistance to *Fusarium *wilt and sterility mosaic disease Plant Breed 2010 129 135 141 10.1111/j.1439-0523.2009.01698.x

[B34] Sivaramakrishnan S Seetha K Rao AN Singh L RFLP analysis of cytoplasmic male sterile lines in Pigeonpea (*Cajanus cajan *L. Millsp.) Euphytica 1997 126 293 299

[B35] Yang S Pang W Harper J Carling J Wenzl P Huttner E Zong X Kilian A Low level of genetic diversity in cultivated pigeonpea compared to its wild relatives is revealed by diversity arrays technology (DArT) Theor Appl Genet 2006 113 585 595 10.1007/s00122-006-0317-z 16845522

[B36] Nayak SN Zhu H Varghese N Datta S Choi H Horres R Jungling R Singh J Kavi Kishor PB Sivaramakrishnan S Hoisington DA Kahl G Winter P Cook DR Varshney RK Integration of novel SSR and gene-based SNP marker loci in the chickpea genetic map and establishment of new anchor points with *Medicago truncatula *genome Theor Appl Genet 2010 120 1415 1441 10.1007/s00122-010-1265-1 20098978 PMC2854349

[B37] Cordoba JM Chavarro C Schlueter JA Jackson SA Blair MW Integration of physical and genetic maps of common bean through BAC-derived microsatellite markers BMC Genomics 2010 11 436 10.1186/1471-2164-11-436 20637113 PMC3091635

[B38] Varshney RK Penmetsa RV Dutta S Kulwal PL Saxena RK Datta S Sharma TR Rosen B Carrasquilla-Garcia N Farmer AD Dubey A Saxena KB Gao J Fakrudin B Singh MN Singh BP Wanjari KB Yuan M Srivastava RK Kilian A Upadhyaya HD Mallikarjuna N Town CD Bruening GE He G May GD McCombie R Jackson SA Singh NK Cook DR Pigeonpea genomics initiative (PGI): an international effort to improve crop productivity of pigeonpea (*Cajanus cajan *L.) Mol Breed 2010 26 393 408 10.1007/s11032-009-9327-2 20976284 PMC2948155

[B39] Beckmann JS Soller M Toward a unified approach to genetic mapping of eukaryotes based on sequence tagged microsatellite sites Nat Biotechnol 1990 8 930 932 10.1038/nbt1090-930 1366775

[B40] Kreike CM Stiekema WJ Reduced recombination and distorted segregation in a *Solanum tuberosum *(2*x*) × *S. spegazzinii *(2*x*) hybrid Genome 1997 40 180 187 10.1139/g97-026 18464817

[B41] Kianian SF Quiros CF Generation of a *Brassica oleracea *composite RFLP map: linkage arrangements among various populations and evolutionary implications Theor Appl Genet 1992 84 544 554 10.1007/BF00224150 24201339

[B42] Elangovan M Rai R Dholakia BB Lagu MD Tiwari R Gupta RK Rao VS Roder MS Gupta VS Molecular genetic mapping of quantitative trait loci associated with loaf volume in hexaploid wheat (*Triticum aestivum*) J Cereal Sci 2008 47 587 598 10.1016/j.jcs.2007.07.003

[B43] Yashitola J Thirumurugan T Sundaram RM Naseerullah MK Ramesha MS Sarma NP Stone RV Assessment of purity of rice hybrids using microsatellite and STS markers Crop Sci 2002 42 1369 1373 10.2135/cropsci2002.1369

[B44] Sundaram RM Naveenkumar B Biradar SK Balachandran SM Mishra B IlyasAhmed M Viraktamath BC Ramesha MS Sharma NP Identification of informative SSR markers capable of distinguishing hybrid rice parental lines and their utilization in seed purity assessment Euphytica 2008 163 215 224 10.1007/s10681-007-9630-0

[B45] Asif M Rahman MU Zafar Y Genotyping analysis of six maize (*Zea mays *L.) hybrid using DNA fingerprinting technology Pakistan J Bot 2006 38 1425 1430

[B46] Ali MA Seyal MT Awan SI Niaz S Ali S Abbas A Hybrid authentication in upland cotton through RAPD analysis Aust J Crop Sci 2008 2 141 149

[B47] Mishra GP Singh RK Mohapatra T Singh AK Prabhu KV Zaman FU Sharma RK Molecular mapping of gene for fertility restoration of wild abortive (WA) cytoplasmic male sterility using a basmati rice restorer line J Plant Biochem Biot 2003 12 37 42

[B48] Nandakumar N Singh AK Sharma RK Mohapatra T Prabhu KV Zaman FU Molecular fingerprinting of hybrids and assessment of genetic purity of hybrid seeds in rice using microsatellite markers Euphytica 2004 136 257 264 10.1023/B:EUPH.0000032706.92360.c6

[B49] Cuc LM Mace ES Crouch JH Quang VD Long TD Varshney RK Isolation and characterization of novel microsatellite markers and their application for diversity assessment in cultivated groundnut (*Arachis hypogaea*) BMC Plant Biol 2008 8 55 10.1186/1471-2229-8-55 18482440 PMC2416452

[B50] Ewing B Green P Base-calling of automated sequencer traces using Phred. II. Error probabilities Genome Res 1998 8 186 194 9521922

[B51] Anderson JA Churchill GA Sutrique JE Tanksley SD Sorrells ME Optimizing parental selection for genetic linkage maps Genome 1993 36 181 186 10.1139/g93-024 18469981

[B52] Lander ES Green P Abrahamson J Barlow A Daly MJ Lincoln SE Newburg L MAPMAKER: an interactive computer package for constructing primary genetic linkage maps of experimental and natural populations Genomics 1987 1 174 181 10.1016/0888-7543(87)90010-3 3692487

[B53] Kosambi DD The estimation of map distance from recombination values Ann Eugen 1944 12 172 175

[B54] Van Ooijen JW Voorrips RE JoinMap 3.0, software for the calculation of genetic linkage maps Plant Research International Wageningen, The Netherlands 2001

[B55] Dettori MT Quarta R Verde I A peach linkage map integrating RFLPs, SSRs, RAPDs and morphological markers Genome 2001 44 783 790 10.1139/gen-44-5-783 11681601

[B56] Voorrips RE MapChart: software for the graphical presentation of linkage maps and QTLs J Hered 2002 93 77 78 10.1093/jhered/93.1.77 12011185

